# The interplay of legal norms in regulating digital platform mergers: comparative insights from China, ASEAN, and the European Union with policy implications for Thailand

**DOI:** 10.3389/fsoc.2026.1917613

**Published:** 2026-08-26

**Authors:** Muhammad Yaseen, Korntima Phattanasin

**Affiliations:** 1 School of International Law, Southwest University of Political Science and Law, Chongqing, China; 2 School of Law, Center of Geosocial and Cultural Research for Sustainable Development (GSCR), Walailak University, Nakhon Si Thammarat, Thailand

**Keywords:** ASEAN regulation, data-driven markets, digital platform mergers, interplay of legal norms, killer acquisitions, non-price competitive factors, socio-legal analysis

## Abstract

The rise of digital platforms has disrupted global markets by creating data-driven business models and strong network effects, challenging the normative structures of traditional merger control frameworks. These technological dynamics have increased the risk of killer acquisitions, where dominant firms absorb innovative start-ups before they evolve into effective competitors. In many emerging economies, the institutional behavior and legal norms underlying merger notification systems rely heavily on traditional turnover thresholds and price-based assessment tools, failing to capture early-stage digital firms with low revenue but high socio-economic impact. Through a comparative analysis of regulatory frameworks in China, selected ASEAN jurisdictions (Malaysia, Indonesia, and Singapore), the European Union, and international guidance from the OECD and UNCTAD, this paper examines how different legal systems respond to data-driven digital mergers and evaluates their relevance for Thailand’s emerging merger-control framework. Thailand is positioned as the central policy case, while other jurisdictions are analyzed as comparative benchmarks representing different levels of regulatory maturity and institutional capacity. The findings reveal a shift in system rationality: advanced jurisdictions have begun integrating non-price factors and more flexible review mechanisms, whereas many developing economies remain bound by traditional, ill-suited normative frameworks. This paper focuses on Thailand as the central policy case, with the aforementioned jurisdictions serving as comparative benchmarks, and provides evidence-based recommendations. To effectively govern digital markets, Thailand must update its legal norms and institutional capacity by incorporating transaction-value thresholds, SSNDQ, data-access analysis, and ex-post review mechanisms to mitigate the disruptive effects of digital concentrations.

## 1 Introduction

Digitalization has transformed the structure and dynamics of global markets, giving rise to platform-based business models that depend on data accumulation, multi-sided user interactions, and algorithmic value creation. These developments have reshaped traditional competition paradigms, making it increasingly difficult for regulators to assess market power, identify anticompetitive conduct, and control mergers effectively. A defining characteristic of digital markets is the prevalence of zero-price services, where users pay not with money but with personal and behavioral data. Combined with indirect network effects, rapid scaling capabilities, and cross-market integration, these features create strong incentives for dominant platforms to expand through strategic acquisitions rather than organic growth (Ong and Toh, 2023; U.S. Department of Justice Antitrust Division and Federal Trade Commission, 2023). This environment has facilitated the emergence of *killer acquisitions*, in which incumbent firms acquire nascent competitors primarily to neutralize future competitive threats, limit innovation, and consolidate data advantages. This study addresses the research question: *How can emerging economies, particularly Thailand, adapt merger control frameworks to effectively detect and regulate killer acquisitions in data-driven digital platform markets?* Thailand is chosen as the primary policy case due to its emerging economy status and ongoing reforms, while China, ASEAN, and the EU serve as comparative benchmarks. The conceptual framework integrates killer acquisitions, platform economics, and non-price competition to guide regulatory evaluation (Hemphill and Wu, 2020; Bryan and Hovenkamp, 2020; Sidak and Teece, 2009).

To clarify the analytical framework, this study conceptualizes digital merger regulation through three interconnected dimensions: legal norms, institutional behavior, and regulatory adaptation. Legal norms refer to the formal rules, principles, and interpretative standards that determine when and how merger transactions are reviewed, including notification thresholds, substantive assessment criteria, and available enforcement powers. In traditional merger-control systems, these norms were largely designed around industrial markets characterized by measurable prices, tangible assets, and observable turnover. However, digital platform markets challenge these assumptions because competitive advantage is increasingly derived from intangible assets, particularly user data, algorithms, network effects, and innovation capabilities.

Institutional behavior refers to the way competition authorities interpret and apply legal norms in practice. In digital markets, regulatory effectiveness depends not only on the existence of legal provisions but also on institutional capacity to identify emerging theories of harm, evaluate non-price competition, analyze data-related advantages, and monitor rapidly changing platform ecosystems. Therefore, differences in regulatory outcomes across jurisdictions are influenced by both legal design and institutional capabilities.

Regulatory adaptation describes the process through which merger-control systems modify traditional approaches to respond to digital-market characteristics. This adaptation involves moving beyond exclusive reliance on turnover thresholds and price-based assessment tools toward more flexible mechanisms incorporating transaction-value indicators, below-threshold review powers, innovation analysis, data-access considerations, and quality-based assessments. The framework adopted in this study therefore evaluates digital merger regimes according to their capacity to capture competitive risks arising from data concentration, network effects, ecosystem expansion, and the loss of potential future competition.

Traditional merger-control instruments remain valuable but may be insufficient when applied alone to digital platforms. Turnover-based thresholds may fail to identify strategically important acquisitions because start-ups often generate limited revenue despite possessing significant data assets, technological capabilities, or future competitive potential. Similarly, price-oriented tests such as the SSNIP framework provide limited guidance in zero-price and multi-sided platform markets where competition occurs through service quality, innovation, privacy protection, user engagement, and ecosystem accessibility. Consequently, this study adopts a broader analytical framework in which competition effects are assessed through five interconnected factors: (1) data accumulation and control, (2) network effects and platform dependency, (3) innovation and potential competition, (4) quality and non-price dimensions of consumer welfare, and (5) institutional capacity to implement effective review mechanisms.

While merger control authorities in advanced jurisdictions have begun adapting to these realities, emerging economies often continue to operate within frameworks designed for traditional, price-based markets (Gürkaynak et al., 2014). Their reliance on turnover thresholds, the SSNIP test, and narrowly defined market-share metrics leads many digital mergers to evade review entirely despite posing significant long-term risks to competition. As a result, there is growing international concern about the capacity of existing regulatory systems to detect anticompetitive consolidation in digital markets, particularly in jurisdictions with developing institutional infrastructures.

This paper contends that traditional merger control tools and thresholds in emerging markets, particularly those based on turnover thresholds and price-based analysis, are not sufficient to control digital market mergers and to identify killer acquisitions. To support this argument, the study uses a comparative analytical approach that focuses on the key regulatory dimensions in the three regions: China, ASEAN member states, and the European Union. The comparative lens is on notification systems, threshold design, assessment methods, non-price effects, incorporation of data-related factors, and the ability to review below-threshold transactions.

This study builds on the previous studies by combining the China, ASEAN, EU, and international institutional perspectives in a single comparative framework for digital-platform mergers. Digital merger regulation should also be understood within the broader context of digital governance and institutional capacity. The effectiveness of regulatory frameworks increasingly depends on how public institutions collect, manage, interpret, and govern digital information. Recent research on digital government and data governance demonstrates that institutional transparency, information availability, and data-management capabilities can influence organizational behavior and regulatory effectiveness. Tian and Wang (2026) show that digital government services can strengthen institutional transparency and improve compliance outcomes by reducing information asymmetry between regulators and regulated entities. Similarly, Wang et al. (2026) highlight that effective government data governance can facilitate organizational upgrading by improving access to information resources and supporting innovation-oriented development (Tian and Wang, 2026; Wang et al., 2026).

These insights are relevant to digital merger control because competition authorities increasingly rely on information-intensive regulatory processes. Assessing platform mergers requires access to reliable market information, technological expertise, and analytical capacity to evaluate data assets, network effects, innovation potential, and ecosystem structures. Therefore, digital merger governance is not only a question of legal rules but also a question of institutional capability to manage and interpret complex digital information environments.

It explains why thresholds based on turnover and tools based on price are not effective in digital markets and highlights the need for data access assessments, SSNDQ analysis, thresholds on transaction value, and ex-post review mechanisms. The paper proposes a novel application of these insights to Thailand and provides a specific policy agenda for enhancing the digital merger regime and combating killer acquisitions.

Recent economic literature on digital platform competition and merger control highlights that acquisitions in digital markets may generate dynamic efficiency losses and reduce innovation incentives, particularly in cases involving nascent competitors and data-driven ecosystems. This study builds on this literature by extending the analysis to emerging economies, with a particular focus on Thailand.

## 2 Methodology

The jurisdictions included in this study are selected purposively to represent contrasting regulatory models and institutional contexts relevant to Thailand’s digital merger-control reform. Thailand is selected as the primary policy case because it represents an emerging digital economy where merger-control mechanisms are developing but face challenges in addressing platform-based transactions, data-driven competition, and acquisitions involving low-revenue but strategically significant firms. The comparative jurisdictions are chosen according to their regulatory relevance rather than geographic completeness.

The European Union is selected as a mature competition-law system because it provides an advanced example of digital merger assessment through the Significant Impediment to Effective Competition (SIEC) framework, consideration of non-price factors, innovation effects, and data-related theories of harm. China is included because it represents a rapidly evolving emerging economy that has introduced platform-specific competition guidelines, below-threshold review powers, and institutional mechanisms designed to address digital-market concentration. Within ASEAN, Singapore, Indonesia, and Malaysia are selected because they demonstrate different stages of merger-control development: Singapore represents a flexible and institutionally developed model, Indonesia illustrates an ex-post notification framework, and Malaysia reflects a jurisdiction currently developing a broader merger-control regime. International organizations, including the OECD and UNCTAD, are incorporated as normative reference points rather than as legal jurisdictions because their materials provide policy guidance on emerging competition-law challenges.

The comparative analysis distinguishes between three categories of regulatory materials. First, binding legal instruments include legislation, regulations, merger-control rules, and formally enforceable decisions issued by competent authorities. Examples include the EU Merger Regulation, China’s Anti-Monopoly Law, Singapore’s Competition Act, Indonesia’s competition legislation, and Thailand’s Trade Competition Act B. E. 2,560 (2017). Second, non-binding interpretative materials include agency guidelines, policy documents, and international recommendations, such as OECD and UNCTAD guidance, which are analyzed as sources of regulatory development rather than enforceable law. Third, proposed reforms and draft amendments are treated separately and are evaluated only as potential future directions rather than existing regulatory obligations.

To improve analytical consistency, this study applies a functional comparative methodology rather than a simple descriptive comparison. Each jurisdiction is assessed according to five analytical dimensions: (1) notification design and jurisdictional thresholds, including turnover, transaction-value, asset-based, hybrid, or discretionary review mechanisms; (2) substantive merger assessment tools, including price effects, quality effects, innovation competition, data-related advantages, and network effects; (3) the ability to capture below-threshold or low-turnover acquisitions involving potential competitors; (4) institutional enforcement capacity, including investigative powers, technical expertise, and administrative feasibility; and (5) suitability for adaptation within Thailand’s legal and institutional environment.

The suitability of foreign regulatory approaches for Thailand is therefore not evaluated by determining which jurisdiction has the strongest competition regime in absolute terms. Instead, the assessment considers whether specific regulatory features can address Thailand’s identified challenges while remaining legally and institutionally feasible. For example, transaction-value thresholds may improve Thailand’s ability to detect high-value acquisitions of low-revenue digital firms, but their implementation requires administrative capacity to evaluate transaction valuation. Similarly, ex-post review powers may increase regulatory flexibility but require sufficient resources for continuous market monitoring. The comparative framework therefore evaluates both regulatory effectiveness and practical implementation constraints.

This methodological approach enables the study to move beyond identifying regulatory differences and instead evaluate which institutional and legal mechanisms are transferable, adaptable, and practically feasible for Thailand’s digital merger-control development.

### 2.1 Thailand as the central policy case: digital merger-control challenges in an emerging economy

Thailand provides the central policy context for this study because its merger-control framework reflects the broader challenges faced by emerging economies attempting to regulate digital-platform consolidation. The Thai merger-control system is established under the Trade Competition Act B. E. 2,560 (2017), which introduced a dual structure consisting of ex-ante approval for mergers that may create a monopoly or dominant position and ex-post notification for mergers that may substantially lessen competition. This structure represents an important development compared with jurisdictions lacking general merger-control mechanisms; however, it was primarily designed around conventional competition concerns rather than the distinctive characteristics of digital-platform markets.

Under Thailand’s current framework, mergers requiring prior approval are assessed for the risk of monopoly creation or dominance, while certain completed transactions must be notified after implementation when they may substantially reduce competition. The system therefore combines preventive and corrective approaches. However, digital mergers create additional regulatory difficulties because competitive significance may not be reflected in immediate turnover, assets, or market share. Digital start-ups may have limited revenue but possess valuable data assets, technological capabilities, user networks, or innovation potential that may influence future competition.

The central challenge for Thailand is therefore not simply the absence of merger-control rules, but the limited ability of traditional legal instruments to capture digital-market dynamics. Existing notification approaches may overlook acquisitions involving early-stage digital firms because their current financial indicators do not reflect their strategic importance. Similarly, conventional market analysis may fail to identify competitive risks arising from data accumulation, ecosystem expansion, algorithmic advantages, and reduced innovation incentives.

Institutional capacity represents another important dimension of Thailand’s digital merger-control challenge. Effective review of platform acquisitions requires competition authorities to evaluate complex economic and technological factors, including network effects, multi-sided markets, data dependency, interoperability barriers, and potential competition. These assessments require specialized expertise and analytical resources beyond traditional merger review.

The comparative analysis therefore uses China, selected ASEAN jurisdictions, and the European Union not as separate case studies of equal importance, but as regulatory reference points for identifying transferable mechanisms that may strengthen Thailand’s framework. The following sections examine which elements of foreign approaches—including transaction-value thresholds, below-threshold review powers, non-price competition analysis, and institutional specialization—may provide feasible solutions for Thailand.

## 3 Regulating mergers and acquisitions in digital markets in China

Under Chapter IV on AML undertakings, the merger control system operates. Article 25 of the AML defines concentration of undertakings as mergers and acquisitions that provide control over target entities through share or asset purchases, or strategic agreements that allow control or substantial market impact.

### 3.1 Data-driven merger

Under the Anti-Monopoly Law (AML), concentration management falls under Articles 25 to 38, while Articles 26–33 establish essential provisions. Pursuant to Article 26, every business that surpasses the Threshold mentioned in the State Council Declaration for Concentration of Undertakings (2018) must file a notification with the anti-monopoly law enforcement agency of the State Council. An agency would need to ask for a declaration when a merger presents possible competition restrictions. Article 27 applies an exclusion when one party holds greater than 50% voting share control of another company or when a single entity outside the merger possesses 50% ownership of all participating businesses. The required documents for evaluation are outlined in Article 28, and the post-merger examination period reaches up to 90 days according to Article 31, whereas Article 30 introduces a preliminary review phase of 30 days. Article 33 details the assessment standards for merger effects. Under pre-transaction notification requirements, AML transactions undergo regulatory review before they can be finalized (Pantazatou et al., 2025). Any company that delivers falsified information must face both monetary penalties and criminal sanctions. The system will cancel a transaction when the authorization occurs, but the information later proves false (Albshaier et al., 2024). The law requires suppliers to provide accurate data, which prevents them from producing false positivity in testing results. Two stages of assessment follow the preliminary 30-day review period, according to Articles 30 and 31. Before getting approval, every transaction remains on hold. Article 33 details merger assessment criteria, including market share, market concentration factors, technical innovation, consumer effects, national economic development indicators, and other factors. Article 33(6) features a broad scope that enables diverse evaluations of mergers through data-based network effect assessments. Article 20 of the Guideline for Online Platform Economy Areas specifies the criteria used for evaluating mergers in online platforms. Market share is measured using key metrics, including transaction volume, clicks, usage duration, and active user count (Qin et al., 2024). The analysis of market leadership for operators includes assessing their influence on the market alongside their ability to retain users while maintaining control of data connections and their special resource access. Market concentration requires analyzing the number of competitors as one of its main components. Market entry becomes a challenge because operators need access to technology and data, channels, intellectual property, and user switching fees. New technology assessments focus on operational effects that acquisitions have on industrial advancements. The review of consumer impact analyzes decreased product quality and selection, discriminatory practices, improper use of customer information, and diminished choices. The AML’s merger control system implements the evaluation methods for data-driven mergers through the Guideline for Online Platform Economy Areas under Article 20, which includes analysis of network effects and user data combination (J. W. Lee, 2023). The AML requires analysis of both pre-merger advertising benefits and post-merger service quality changes. As demonstrated in Qihoo v., the competitive pressure evaluation in free online services utilizes the SSNDQ test (Small but Significant Non-transitory Decrease in Quality) according to paragraph (6) (Qihoo 360 and Tencent, 2013; Nademi, 2025). An analysis of service quality changes after mergers belongs to the merger quality dimension of the investigation. Consumer impacts from the merger are discourteous because users possess alternative service options. This leads to an absence of concentration.

### 3.2 Killer acquisition

Under China’s Anti-Monopoly Law (AML), a merger declaration serves as the core regulatory element. The State Administration for Market Regulation (SAMR) mandates that companies seek approval before completing a merger, particularly if they exceed turnover thresholds specified in Article 26. If a merger or acquisition falls outside the thresholds but raises concerns about competition restriction, SAMR has the authority to initiate a review. This flexibility allows SAMR to investigate mergers that would otherwise evade scrutiny under the current thresholds.

The Regulation on Declarations Standard for Concentration of Undertakings specified the turnover thresholds that applied prior to the 2024 reform (in force from 2008 until January 2024). These were:

- A combined global turnover exceeding RMB 10 billion (approximately USD 1.4 billion) with at least two parties each generating more than RMB 400 million (approximately USD 56 million) in the preceding fiscal year, or
- A combined turnover in China exceeding RMB 2 billion (approximately USD 280 million), with at least two companies each generating more than RMB 400 million (approximately USD 56 million) in Chinese revenue in the preceding year (State Council of the People’s Republic of China, 2018).

While traditional turnover thresholds may miss high-impact acquisitions, SAMR’s below-threshold call-in authority allows review of deals raising competition concerns. The 2024 threshold reforms raise filing requirements to combined worldwide turnover above RMB 12 billion or China turnover above RMB 4 billion, with at least two parties generating RMB 800 million each. This demonstrates that call-in powers and non-price analysis (network effects, data access, innovation potential) are critical to capture high-impact below-threshold acquisitions, as reflected in China’s 2024 merger filing threshold reforms and SAMR’s retained discretion to call in transactions with competitive concerns (Xiong et al., 2025; Zou et al., 2025).

This change addresses the problem of acquisitions in the digital economy, where start-ups with limited revenue but significant market potential are often acquired by larger incumbents in killer acquisitions. These reforms bring China’s regulatory framework closer to international standards, aligning more with approaches seen in the EU and US, which are increasingly focused on transaction value and market impact rather than just turnover.

This concern is widely supported in the industrial organization literature, which shows that incumbent firms may acquire potential competitors to eliminate future innovation threats rather than to achieve immediate cost efficiencies. Such “killer acquisitions” can reduce long-term innovation incentives and weaken competitive pressure in platform markets (Bourreau and De Streel, 2020; Cabral et al., 2021). Moreover, theoretical work demonstrates that merger control policies may have unintended effects on innovation strategies and entry behavior, highlighting the need for forward-looking regulatory frameworks (Benkert et al., 2025).

### 3.3 Lessons for Thailand from China

China’s experience provides three important lessons for Thailand. First, the use of below-threshold review powers demonstrates the importance of maintaining regulatory flexibility where digital acquisitions involve firms with limited current revenue but significant future competitive potential. Second, China’s incorporation of data control, network effects, user engagement, and innovation factors into platform-merger analysis illustrates the need for Thailand to expand beyond traditional price and turnover-based assessment. Third, China’s institutional approach highlights that legal reform must be accompanied by administrative capacity because digital merger review requires specialized expertise in technology-driven markets.

## 4 Regulating mergers and acquisitions in digital markets in ASEAN

### 4.1 Data-driven merger

#### 4.1.1 Competition law of Malaysia

The current Competition Act 2010 of Malaysia lacks regulations for merger-related matters. The Malaysian Aviation Commission Act of 2015 applies merger control only to aviation, while the Communications and Multimedia Act of 1998 applies it to communications and multimedia (Rahman et al., 2024). The absence of merger and acquisition regulation in the Competition Act of 2010 requires combined business owners to exercise caution, as their activities may violate multiple provisions of the Act, including dominance abuse. Malaysia’s present competition law lacks merger prohibition provisions, which prevent its intervention in deals that cause a substantial reduction in market competition (C. Lee, 2014). Businesses refrain from legal intervention for cartels after using mergers as collateral because there currently exist no provisions in Malaysia’s competition law to stop them. Changes to the Competition Act 2010 continue to advance at the Malaysian Competition Commission (MyCC). A general merger control system forms part of these reforms, which prohibits mergers or anticipated mergers that might diminish market competition in any Malaysian sector. The proposed merger control reform leaves undefined the specific benchmarks to evaluate merger effects through its two assessment stages. A general assessment of the merger transaction is performed in the preliminary review stage. The agencies conduct a more thorough evaluation when they determine that a merger holds significant anticompetitive power in the market.

#### 4.1.2 Competition law of Indonesia

The legal authority for mergers and acquisitions transactions in Indonesia appears in Law No. 5 of 1999 through Articles 28 and 29. Additional provisions exist within Government Regulation No. 57 of 2010 (GR No. 57/2010) and KPPU Regulation No. 3 of 2023, which extended the provisions first published in 2019. Under Article 29 of Indonesia’s Law No. 5 of 1999, companies first need to notify KPPU about their transactions after the deal completes (ex-post approach) when their sales or assets exceed the limitations set by GR No. 57/2010. Article 28 bans monopolistic conduct and unfair competition that occurs through mergers, consolidations, or acquisitions. The notification process, together with supporting competition and impact assessment documents, becomes mandatory under Article 2(2) of KPPU Regulation No. 3/2023 within 30 days from the legal effective date.

To submit a merger notification, authorities need an official form indicating the merger type, supporting documentation, identification, and an explanatory letter. Organizations should submit financial records from the previous 3 years, along with their corporate framework before and after the merger, detailing their post-transaction strategy, providing market share estimates, and identifying impacted regions with expected benefits as supporting evidence. The KPPU assesses the submission by analyzing the submitted materials and making presumptions, supported by additional information gathered (Eduardo et al., 2025). The procedure starts by identifying targeted markets before assessing pre- and post-merger market concentration patterns and analyzing entry barriers and anticompetitive impacts. Interesting to note. The KPPU then conducts assessment-stage reviews for every transaction showing potential anticompetitive effects during its initial evaluation phase. Mergers or acquisitions receive their first examination through the preliminary assessment process. Indonesia’s compulsory post-merger notification requirement reduces false-positive merger reviews. Businesses operating in Indonesia or abroad must contact KPPU within 30 days after a merger is completed so KPPU can review actual results rather than projections. The review system includes two phases: an initial review and a more detailed review if the assessment reveals possible competitive risks. The assessment moves to a commissioner panel for extended evaluation whenever KPPU detects potentially dangerous circumstances according to KPPU Regulation No. 3/2023. The evaluation procedure according to Regulation No. 3/2023 Article 22(2) includes a detailed examination of any potential unfair business activities. Article 22(3) maintains that “The Commission determines the analysis method as intended in paragraph (2).” Under Article 22(3), the Commission retains flexible discretion because of its broad analysis language, although its method remains undefined. The Commission appears to plan on utilizing data-driven effect variables during evaluations when it determines the presence of data-driven network effects within the merging or acquired platforms.

#### 4.1.3 Competition law of Singapore

The Competition Act 2004 (the Act) prohibits mergers that create or enhance a substantial lessening of competition (SLC) in any market in Singapore, whether the merger takes place in Singapore or elsewhere. According to Section 54 of the Act, mergers that create or enhance a substantial lessening of competition (SLC) in any Singaporean market are prohibited, whether the merger occurs in Singapore or elsewhere. The Act introduces a voluntary notification system, under which parties are not obligated to notify the Competition and Consumer Commission of Singapore (CCCS), but may do so to avoid later infringing the Act. CCCS can also terminate a merger under Section 58A if it believes that the merger is likely to violate Section 54.

In assessing mergers, CCCS applies its *Guidelines on the Substantive Assessment of Mergers*, which consider market shares, concentration levels, direct and indirect network effects, entry barriers, data-related advantages, countervailing buyer power, and risks of coordinated behavior (Valiyeva, 2024). There is no fixed threshold for determining SLC; instead, CCCS conducts a case-by-case analysis, including counterfactual assessment, to evaluate whether a merger would harm competition through higher prices, reduced quality, diminished innovation, or exclusion of rivals. This approach is particularly important in data-driven markets where price effects alone may not reveal competitive harm.

The Grab–Uber merger illustrates Singapore’s enforcement approach. Although the parties did not notify CCCS, the authority initiated an investigation in 2018 after the transaction gave Grab an estimated 80% market share. CCCS found that the merger raised barriers to entry through exclusivity arrangements and strengthened network effects. As a result, CCCS imposed SGD 13 million in fines and required Grab to remove exclusivity clauses and maintain pre-merger pricing algorithms. This case demonstrates that, despite its voluntary regime, Singapore can effectively address anticompetitive digital mergers through ex-post intervention.

### 4.2 Killer acquisition

#### 4.2.1 Competition law of Malaysia

Merger-regulation specifications do not exist in the Malaysian Competition Act 2010. The Malaysian Competition Commission (MyCC) introduced a proposed notification system that includes compulsory and voluntary elements (Zakaria et al., 2025). Both mandatory and optional alert functions will operate within this system when successfully implemented. The Malaysian Competition Commission requires mandatory notification for prescribed threshold-exceeding anticipated mergers that need MyCC approval before the transactions can be finalized. The merger will become invalid because it failed to meet the necessary notification requirements (Choe and Shekhar, 2010). As a default option, companies can provide MyCC with merger or projected merger notices either in advance or following completion of the transaction. The voluntary notification system serves mergers or projected mergers that have no impacts beyond threshold requirements. Under this approach, the merger review notification procedures of the Competition Act of 2010 will be controlled by thresholds. The threshold in question proves inaccurate because the proposed amendment remains active in development. The turnover amount, along with the final business operations, defines the points at which notification must be given. MyCC must establish the notification threshold through an official order, which will be published in the Gazette after the modification of the Competition Act becomes law. MyCC maintains the power to verify and alter the notification threshold by issuing Gazette-published orders.

#### 4.2.2 Competition law of Indonesia

Companies must notify the KPPU regarding mergers and acquisitions of specified assets or sales value levels according to Article 29(1) of Law No. 5 of 1999. The law, through Article 28, requires merger notifications when the combination may cause a monopoly or unfair competition.

Article 10 of GR 57/2010 provides companies with the choice of pre-merger notification, which allows them to seek KPPU consultation before executing mergers. Merger notification remains compulsory after the deal, yet collaboration with KPPU through pre-notification helps companies avoid anti-competitive problems and provides legal certainty.

The following requirements for notification are spelled out in GR 57/2010:

1. The company operates with assets exceeding IDR 2.5 trillion, equivalent to USD 170 million (Sukarmi et al., 2024).
2. The market in Indonesia achieved IDR 5 trillion in sales, which corresponds to USD 340 million.

Controlling killer acquisitions proves difficult because most start-ups fail to meet these regulatory thresholds. The existing framework of Indonesia’s merger control system lacks adequate measures to address this problem unless authorities introduce modifications.

#### 4.2.3 Competition law of Singapore

Under the Competition Act of 2004, mergers include three specific conditions: Different businesses merge when they unite as separate entities, or when one entity dominates another, or when an organization acquires the substantial assets of another organization. Under the Competition Act 2004 of Singapore, mergers require voluntary notification, but both turnover limits and reporting obligations are absent in this framework. Under Article 58(1), businesses must file a notification only to avoid possible Article 54 violations, and Article 57(1) allows them to submit notice to the Commission before an evaluation. A merger evaluation depends mainly on factual and legal elements to determine control as one of its core points for analysis. Evaluations take place in terms of both de facto and legal aspects. The CCCS guidelines imply that mergers typically avoid competition issues except when the merged entity holds 40% market share or more, or reaches 20–40% market share, or reaches 70% market share when the three biggest companies consolidate their outputs post-merger. The CCCS does not perceive mergers as harmful unless they lead to substantial market concentration growth (Koirala et al., 2023). The merger control framework of the CCCS operates freely without turnover requirements to achieve effective monitoring of business acquisitions involving digital startups, along with significant acquisitions, without imposing rigid constraints.

This comparison illustrates that low-turnover, high-impact acquisitions are often missed, particularly under Indonesia’s ex-post notification system and Malaysia’s incomplete framework. Supplementary mechanisms such as transaction-value thresholds and non-price assessments are necessary to address digital-market risks.

### 4.3 Lessons for Thailand from selected ASEAN jurisdictions

The ASEAN comparison demonstrates that Thailand faces challenges shared by other emerging digital economies but also provides practical models for reform. Singapore illustrates the value of flexible substantive assessment and the ability to examine non-price competitive effects, including quality, innovation, and network effects. Indonesia highlights the limitations of relying primarily on ex-post notification because completed digital acquisitions may become difficult to reverse after-market structures have changed. Malaysia demonstrates the importance of developing a comprehensive merger-control framework capable of addressing digital-sector transactions. For Thailand, these experiences suggest that regulatory reform should focus not only on thresholds but also on institutional readiness, investigative flexibility, and analytical capacity.

## 5 Regulating mergers and acquisitions in digital markets in the EU

Council Regulation (EC) No. 139/2004 from January 20, 2004, establishes the EU Merger Regulation that forms the basis of EU merger control. The legislation defines a merger or acquisition that causes market concentration as a “concentration”.

### 5.1 Data-driven merger

Under Article 4(1) of the EU Merger Regulation, mergers with a community dimension must be notified before implementation, with Article 1 establishing mandatory turnover thresholds. Once notified, the European Commission or a relevant Member State assesses whether the concentration may impede effective competition, following the *Guidelines on the Assessment of Horizontal Mergers*. A central part of this assessment is determining whether the merger strengthens dominance, enables price increases, or reduces quality, choice, or innovation.

Traditional tools such as the SSNIP test help measure market power, but they are unsuitable for zero-price digital markets (Mandrescu, 2018). To address this gap, the Commission applies the SSNDQ test to evaluate quality effects, as illustrated in cases such as *Microsoft/Skype*, where non-price factors played a crucial role. EU merger control, therefore, treats non-price harms, quality, innovation, privacy, and choice as equally important to price effects.

The guidelines further require analysis of market shares, entry barriers (including access to data and IP), countervailing buyer power, and risks of coordinated behavior. In digital markets, data access has emerged as a critical competitive parameter (Fast et al., 2023). Cases such as *Google/Fitbit* and *Google/DoubleClick* show the Commission’s concern that merging entities may combine datasets to enhance targeting capabilities, entrench dominance, and raise entry barriers for rivals. However, the EU’s ex-ante framework may overlook risks when mergers involve small target firms whose data value is not yet visible. The *Facebook/WhatsApp* case illustrates this limitation: Facebook assured the Commission that it could not technically match WhatsApp user data with its own systems, yet later implemented such integration, resulting in a €110 million fine for providing misleading information. The case highlights the challenges of assessing data-driven mergers when key data practices may evolve after the transaction.

### 5.2 Killer acquisition

EU merger control assesses non-price harms such as innovation loss, data concentration, and reduced choice. Following the CJEU Illumina/GRAIL judgment, Article 22 can no longer serve as a broad tool for below-threshold acquisitions, highlighting reliance on national jurisdictional hooks (Illumina Inc. and Grail Inc, 2024; Lindeboom, 2025). EU experience demonstrates that transaction-value thresholds, data-access assessment, and innovation analysis are essential for detecting digital-market harms (Table 1).

**Table 1:** Comparative approaches to killer acquisition review.

Jurisdiction	Primary mechanism to capture killer acquisitions	Specific thresholds or criteria	Effect/rationale
Germany	Transaction Value Threshold (TVT)	One party’s domestic turnover > EUR 50 million and target domestic turnover < EUR 17.5 million (Section 35(1a))	Captures low-revenue, high-value start-ups by focusing on the large size of the overall transaction value.
China	Market Value Criterion (Proposed) / Call-in Power	Draft proposal uses a target market valuation of at least RMB 800 million as an alternative to turnover. Currently relies on SAMR’s Article 26(2) power to review below-threshold deals.	Addresses the inadequacy of traditional turnover thresholds in reflecting the competitive potential of nascent digital firms.
European Union (EU)	Turnover thresholds under Article 1 EUMR, supplemented only residually by Article 22 referrals where a Member State has national jurisdiction or a valid call-in power	EU dimension is based on Article 1 turnover thresholds (e.g., combined worldwide turnover above €5 billion and EU-wide turnover of at least two firms above €250 million, or the alternative Article 1(3) thresholds). After the CJEU’s 3 September 2024 *Illumina/GRAIL* judgment, Article 22 cannot be treated as a general mechanism for reviewing transactions that fall below both EU and national thresholds.	The EU has a sophisticated framework for assessing non-price harms, innovation loss, and data effects. But after the CJEU’s Illumina/GRAIL judgment, Article 22 of the EU Merger Regulation can no longer be used to review deals falling below both EU and national thresholds where the referring Member State lacks its own jurisdiction. Capturing below-threshold digital acquisitions now depends largely on national mechanisms, transaction-value thresholds, and other targeted tools.
United Kingdom (UK)	Hybrid / Acquirer Threshold	The acquirer must have a UK turnover of> £350 million and a share of supply/purchase of 33% or more in the UK, provided the target has a connection to the UK.	Specifically captures vertical and conglomerate acquisitions by large incumbents with significant market status, even if the target has minimal UK revenue.
Thailand	Existing Merger Control	Not explicitly detailed	Emerging economy exploring whether existing turnover/asset thresholds are sufficient or if specialized provisions are required.

Source: Author’s compilation based on Germany’s Act Against Restraints of Competition, Section 35(1a); OECD guidance on start-ups, killer acquisitions and transaction-value thresholds; China’s Anti-Monopoly Law and 2024 merger filing threshold reforms; EU Merger Regulation Article 22 and the post-Illumina/Grail limitation on below-threshold referrals; the UK Digital Markets, Competition and Consumers Act 2024; and Thailand’s Trade Competition Act B. E. 2,560 (2017). The threshold mechanisms presented in this table represent different regulatory approaches and should not be treated as equivalent. Turnover thresholds measure the economic size of merging parties based on revenue. Transaction-value thresholds consider the value paid for an acquisition and are designed to capture cases where the target generates limited revenue but possesses significant competitive potential. Market-value criteria refer to the valuation of a company or asset and must be distinguished from turnover-based requirements. Hybrid tests combine multiple indicators, such as acquirer turnover, target activity, market presence, or transaction value. Discretionary call-in powers allow authorities to review transactions that fall below ordinary thresholds where competition concerns exist.

### 5.3 Lessons for Thailand from the European Union

The European Union provides Thailand with important lessons regarding substantive merger assessment. The EU experience demonstrates that digital merger review requires consideration of non-price factors, including innovation, quality, consumer choice, data accumulation, and access conditions. However, the EU experience also shows that even advanced systems face challenges in capturing below-threshold digital acquisitions, particularly after limitations on Article 22 EUMR following the Illumina/GRAIL judgment. For Thailand, the implication is that no single mechanism is sufficient; effective regulation requires a combination of appropriate notification thresholds, flexible review powers, and institutional expertise.

## 6 Regulating mergers and acquisitions in digital Markets in International Institutions

### 6.1 Data-driven merger

#### 6.1.1 OECD

The existing assessment methods under merger control frameworks of multiple jurisdictions face complications due to data-centric digital business combinations. Traditional factors such as market share become insufficient for post-merger impact assessment on free sides of online platforms since traditional methods only examine one market sector (Katagar, 2024). The ex-ante review approach produces erroneous merger assessment positive outcomes due to its forecast nature that predicts possible negative effects before their appearance. Ex-post review stands as a necessary tool for evaluating actual post-merger outcomes, according to the OECD, and France is considering implementing this approach currently. The FTC or DoJ possesses the power to conduct evaluations of past mergers even after they finish, which could lead to multiple enforcement actions against completed deals (Mommers and Landry, 2024). Perform a test to determine if the merger breaches any combination of Section 1 and Section 2 of the Sherman Act and Section 5 of the FTC Act, and Section 7 of the Clayton Act. The FTC seeks information on hundreds of acquisitions performed by major web companies through ex-post assessment of past mergers that have spanned 10 years. According to the OECD, it is necessary to update existing merger notification procedures to resolve digital market merger control issues (Iwuji, 2025). The focus of merger assessment should aim at either individual industries or companies, or adopt an inclusive approach to identify unregulated, potentially harmful transactions. Concern about digital economy merger regulation has escalated among policymakers, together with competition regulators.

The OECD has provided various suggestions and approaches as follows:

1. Market authorities should conduct enhanced checks of digital sector merger and acquisition deals because of fast technological developments and their competitive implications.
2. Assess innovation changes resulting from the merger by analyzing possible competition challenges that exceed basic market share metrics within digital markets.
3. The evaluation process for mergers should include examining data-related issues that involve controlling data amounts and protecting privacy standards and algorithm operations.
4. International competition authorities should unite to address the universal problems developed by digital cross-border mergers through information cooperation.
5. Ex-post review mechanisms should become a possibility to allow regulatory bodies to inspect finalized mergers in case competition problems arise after merger completion.

This perspective aligns with recent economic analyses suggesting that merger control in digital markets must incorporate innovation-based and dynamic efficiency considerations, particularly in cases involving platform ecosystems and data-driven competition (Bourreau and De Streel, 2020).

#### 6.1.2 UNCTAD

The guidelines for mergers control appear within Chapter VI of UNCTAD’s model competition laws. Before implementation, anticipated market-concentrating mergers relying on high market shares after completion must undergo notification. Merger control analysis focuses on several key components as part of its evaluation process. Merger evaluations consist of defining relevant markets together with analyzing competitive products, followed by market share determination and entry barrier assessment (Kaplow, 2021). According to the UNCTAD report, it is essential to adjust antitrust regulations for online markets, yet the report lacks specific solutions to prevent data-based mergers. Digital platforms gain dominance by controlling data while making entry into their operations difficult compared to standard price and market share antitrust investigations (Parker et al., 2020). To protect fair competition within the digital market, frameworks need to amend consumer welfare standards to include factors like choice, privacy, data safeguards, and innovation-based considerations.

### 6.2 Killer acquisition

#### 6.2.1 OECD

Low revenue of new businesses in their beginning phase avoids competition authority review since the set turnover threshold remains too high. The merger control systems of many jurisdictions operate through a notification mechanism based on turnover thresholds to perform merger review either voluntarily or by mandatory regulation (Völcker, 2024). Competitive regulators examine how the merger of these companies would influence market control while decreasing competitive pressure. The merging process can proceed without government examination if the entity surpasses the specified threshold before the merger regulation, while its turnover remains beneath the threshold value. The OECD states that startup companies have issues with merger reviews, which use the turnover figures of combining firms as their main assessment tool (Lee and Geum, 2023). Startup companies generally focus their efforts on building user count and data collection while conducting R&D and data analysis because they need to develop their operations. These companies dedicate more attention to their business initiatives than to profits earned through product sales. The requirement of turnover for inspections makes competition authorities unable to detect large corporations acquiring start-ups since inspections are conditioned on substantial revenue thresholds. The very low start-up turnover made it impossible for competition authorities to detect possible anticompetitive effects of these acquisitions (Bryan and Hovenkamp, 2020). The OECD research shows that authorities have no power to inspect mergers that fail to hit their prescribed turnover limits, enabling parties to proceed with acquisitions without additional examination. The competition legislation specifies limits beyond which regulators lack authority to evaluate mergers. The document indicates that competition regulatory bodies face uncertainty regarding their ability to analyze risky mergers that involve turnover thresholds. Does the existing threshold standard accomplish its goal of detecting anticompetitive mergers, therefore necessitating modification of the turnover criterion for monitoring merger examinations? The research wants to understand if notification system rules prevent review or if review decisions led to the situation. The document proposes that governments should modify their merger review thresholds to use transaction valuations instead of turnover calculations or alternative metrics. This recommendation bases its approach on understanding that particular business sectors need criterion changes, which the existing turnover threshold may not sufficiently identify all applicable transactions. The OECD proposes altering traditional merger control requirements through amendments, which would include both smaller startup acquisitions and innovative target acquisitions to enhance merger control effectiveness, according to their report, which contains many other recommendations to address killer acquisitions. The proposed limit reduction for present turnover stands as one recommendation, yet such a modification would generate extensive mandatory notification for small-value deal transactions. The approach did not receive the needed approval due to its implementation. Several jurisdictions now enforce supplementing value-based thresholds alongside notice requirements for particular corporations. They also have extra thresholds in effect. These supplementary criteria would support investigations of high-value rare transactions that could harm future market competition, along with the existing turnover measures. Strong competitive forecasts relate to present turnover better than current turnover, so these elements should be combined in a changed screening method. The screening approach benefits the market by understanding that losing substantial competitors in future periods has equal destructive potential to existing major competitors. Even though the filter fails to fully capture potential competition losses, it supports turnover as an essential tool to evaluate transactions that decrease market competition in less important markets.

#### 6.2.2 UNCTAD

Acquisitions made by big companies to suppress competition through the purchase of small inventors are recognized as “killer acquisitions.” UNCTAD reports that most merger control systems fail to account for data importance in digital merger evaluations, so they use turnover-based notification criteria instead. Most organizations that possess significant value do not satisfy the turnover Article threshold in targets (Jensen, 2010). Germany implemented a notification requirement, which states in Section 35(1)(a) of the GWB, which requires that mergers be reviewed when the acquisition reaches or exceeds EUR 50 million. The UNCTAD paper recommends that developing countries study German competition law regarding turnover threshold modifications to resolve start-up issues in killer acquisitions. This proposal shares similarities with the OECD.

## 7 Comparing Thailand and other entities

The comparative framework requires updating in light of recent legal developments. In the European Union, the CJEU’s judgment in *Illumina/GRAIL* significantly narrowed the use of Article 22 EUMR, meaning that it can no longer be described as a general route for bringing below-threshold transactions before the Commission where the referring Member State lacks jurisdiction under national law (Tzanaki, 2024). In China, the merger filing thresholds were amended in 2024, and a filing is now generally required where the parties’ combined worldwide turnover exceeds RMB 12 billion or their combined turnover in China exceeds RMB 4 billion, provided that in either case at least two parties each generated more than RMB 800 million in turnover in China in the preceding fiscal year; the RMB 800 million figure is therefore a turnover threshold, not a market-value criterion. In the United Kingdom, merger jurisdiction should be analyzed through the share-of-supply framework and the newer hybrid threshold, rather than any separate “employee share requirement.” For Thailand, a more precise application is necessary because the Trade Competition Act adopts a dual structure consisting of pre-merger approval for mergers that may create monopoly or dominance and post-merger notification for mergers that may substantially lessen competition, with notification due within 7 days after completion and pre-merger review generally completed within 90 days, extendable by 15 days (Table 2).

**Table 2:** Comparative digital merger notification and control frameworks across selected jurisdictions.

Jurisdiction	Notification type	Threshold criteria	Main test applied	Unique features/observations
China	Mandatory (ex-ante)	Turnover thresholds: combined worldwide turnover above RMB 12 billion or combined China turnover above RMB 4 billion, with at least two parties each having more than RMB 800 million turnover in China	Competition-effects/market-impact assessment, including market share, concentration, data control, network effects, entry barriers, innovation, and consumer impact	RMB 800 million is a turnover threshold, not a market-value criterion. China also retains below-threshold review/call-in power where competition concerns arise.
Indonesia	Mandatory (ex-post)	Assets or sales in Indonesia: combined assets above IDR 2.5 trillion or combined turnover above IDR 5 trillion; filing generally due within 30 business days after legal effectiveness	Monopoly/unfair competition assessment, including market concentration and competitive effects	Review occurs after closing, with a review period that can run up to 90 business days; this structure may miss low-turnover digital targets even though it captures completed deals.
Singapore	Voluntary	No mandatory turnover threshold; CCCS uses a voluntary regime with indicative market-share/concentration screens in its guidelines	Substantial Lessening of Competition (SLC)	Flexible, case-specific analysis; CCCS expressly considers price, quality, choice, and other competitive effects, which makes the framework adaptable to digital markets.
EU	Mandatory (ex-ante)	Article 1 EUMR turnover thresholds for concentrations with an EU dimension	Significant Impediment to Effective Competition (SIEC), with strong attention to non-price harms such as innovation, quality, and data-related effects	The EU remains substantively advanced, but after the 3 September 2024 *Illumina/GRAIL* judgment, Article 22 can no longer be treated as a general below-threshold catch-all where the referring Member State lacks national jurisdiction; the Commission later withdrew its 2021 Article 22 guidance.
Thailand	Dual system: mandatory ex ante approval and mandatory ex post notification	Pre-merger approval for mergers likely to create monopoly or dominance; post-merger notification for mergers that may substantially lessen competition and meet the statutory thresholds; post-merger filing is due within 7 days after completion	Monopoly/dominance and substantial-lessening-of-competition framework, rather than a simple SSNIP-only model	Thailand’s regime is more complex than the current table suggests it combines pre-merger approval, 7-day post-merger notification, and a 90-day review period extendable by 15 days for approval cases. Recent OECD review also notes limits in the ex-post branch because the TCCT has no powers over transactions notified only after closing.

Source: Author’s compilation based on China’s Anti-Monopoly Law and merger notification thresholds; Indonesia’s Law No. 5 of 1999, Government Regulation No. 57/2010, and KPPU merger notification rules; Singapore’s Competition Act 2004 and CCCS merger assessment guidelines; EU Merger Regulation No. 139/2004 and EU merger-control practice on non-price effects; Thailand’s Trade Competition Act B. E. 2,560 (2017); OECD competition-law peer review of Thailand; and ASEAN competition-law country materials.

### 7.1 Data-driven merger

Digital mergers require a more holistic analysis than price-based analysis. Competition in zero-price and multi-sided platform markets can be more about quality, innovation, interoperability, access conditions and control over data than about price changes in the moment. In this context, SSNDQ should not be understood as a replacement for all traditional merger-analysis methods, but rather as a complementary analytical tool for markets where competitive harm may occur through quality deterioration rather than price increases. A decrease in quality may include reduced functionality, weaker privacy protection, lower service reliability, reduced innovation incentives, or diminished consumer choice. Therefore, digital merger assessment requires a combination of price-based and non-price indicators depending on the characteristics of the relevant market. It would be more correct to say that the SSNIP test is not sufficient in digital markets, but not irrelevant. A more appropriate comparison is to recognize that price-based instruments can be used as an adjunct to non-price analysis where platform services are offered free of charge or where the risk of competitive harm is more likely to lie in the quality of the services, in the level of innovation, or in the choice of services.

Against that background, the European Union and China presently provide more developed approaches to data-driven mergers than a narrow price-centered model (Pehlivanli, 2025). In the European Union, the substantive assessment under the SIEC standard clearly extends to non-price dimensions of competition, including quality, variety, innovation, and the protection of personal data and privacy. In digital and technology mergers, the Commission’s practice has also examined data-combination effects, access degradation, innovation competition, and long-term entry deterrence. China has likewise moved beyond a purely price-based framework in platform markets by incorporating platform-specific concerns into merger review, including the competitive significance of data, network effects, and market-access conditions. Singapore also offers a comparatively flexible model: although its notification regime is voluntary, CCCS applies a substantially lessening of competition test, uses market-share thresholds only as indicative screens, and may investigate mergers on its own initiative where there are reasonable grounds for concern.

Thailand has a dual merger control regime, under Sections 51 and 52 of the Trade Competition Act, with pre-merger approval for transactions that may lead to monopoly or dominance, and post-merger notification for mergers that may substantially lessen competition, with approval cases typically being reviewed within 90 days, extendable for a further 15 days as necessary. The current system, however, is not as specifically designed to address the realities of the digital market as the comparator jurisdictions referred to above. The Thai regime, in particular, has not yet established a similarly strong framework to assess data collection, ecosystem impacts, theories of harm for innovation, or other non-price theories of harm in platform mergers. This restriction is particularly significant because digital acquisitions may not always be reflected in traditional measures but may create competitive challenges. The EU, China, and Singapore are therefore better prepared than Thailand to systematically deal with mergers involving complex data.

This limitation is also institutional rather than merely doctrinal. The Trade Competition Commission of Thailand (TCCT) has the formal responsibility for supervising merger control, but digital-platform mergers require expertise that is more complex than conventional market-share or turnover assessments. In particular, the TCCT must be able to evaluate network effects, data accumulation, algorithmic advantages, ecosystem dependency, and innovation-based competitive harm. These factors are difficult to assess where the target firm has limited current revenue but strong future competitive significance. As a result, Thailand’s central challenge lies in the gap between the formal legal structure of the Trade Competition Act and the practical enforcement capacity needed to identify low-revenue but high-impact digital acquisitions.

This wording is aligned with the OECD’s caution that SSNIP/SSNDQ should support, not exhaust, digital-market analysis; the European Commission’s explicit treatment of non-price competition under the SIEC test; the Commission’s digital-merger practice on data, access, innovation, and entry deterrence; Singapore’s SLC-based voluntary regime with own-initiative investigations; and Thailand’s dual structure under Sections 51–52 with 7-day post-merger notification and a 90-day review period extendable by 15 days (Trade Competition Act B. E. 2,560 2017).

### 7.2 Killer acquisition

Killer acquisitions remain difficult to capture where merger control relies mainly on turnover-based jurisdictional thresholds, because many digital or innovation-driven targets generate limited current revenue even though they may possess significant future competitive potential. For that reason, the main policy problem is not simply that thresholds are “high,” but that turnover alone may be a poor proxy for competitive significance in digital markets. OECD work has therefore highlighted the limits of turnover-based screening and has pointed to supplementary approaches such as transaction-value thresholds, call-in powers, and other targeted jurisdictional mechanisms for acquisitions involving nascent firms.

In the European Union, concentrations with a Union dimension are still defined primarily by the turnover thresholds in Article 1 EUMR and, following the CJEU’s 3 September 2024 judgment in *Illumina/GRAIL*, Article 22 can no longer be treated as a general tool for reviewing transactions that fall below both EU and national jurisdictional thresholds where the referring Member State lacks competence under its own law. In China, the 2024 reforms raised the merger filing thresholds to combined worldwide turnover above RMB 12 billion or combined China turnover above RMB 4 billion, with at least two parties each having more than RMB 800 million turnover in China; accordingly, RMB 800 million should be understood as a turnover threshold, not a market-value criterion (Hofman and Petry, 2025). At the same time, China retains below-threshold call-in authority, which makes its framework more adaptable than a purely rigid threshold model.

The chosen Asian comparators do not have any compulsory thresholds for turnover in a voluntary merger regime, but CCCS can investigate mergers that result in or are likely to result in a substantial decrease in competition. Indonesia, on the other hand, continues to use the ex-post notification system once the thresholds for assets or turnover are reached, which means that acquisitions with low turnover may not be detected. Thailand is also structurally limited in this respect. It currently has a dual system of pre-merger approval of monopoly- or dominance-creating mergers and post-merger notification within 7 days of the merger of mergers which may substantially lessen competition, with pre-merger approval cases reviewed within 90 days, extendable by 15 days. The Thai framework, however, is not as well-suited to some low-reward, high-stakes acquisitions in fast-moving platform markets, as it still needs acquisitions to reach a threshold to enter the system and does not yet have a well-defined transaction-value trigger and a clear digital-merger call-in model. Malaysia is also an incomplete comparator as the general merger control regime is still in its infancy and does not yet offer a comprehensive economy-wide merger regime.

### 7.3 Empirical illustrations of digital merger effects

Although this study is primarily doctrinal and comparative in nature, selected merger cases are included as illustrative case studies to demonstrate how digital-platform transactions create regulatory challenges that cannot always be captured through traditional merger-control tools. These cases were selected purposively because they represent different dimensions of digital competition concerns, including platform consolidation, data accumulation, ecosystem expansion, and the difficulty of assessing potential competition.

Each case is analyzed according to six dimensions: (1) the competitive relationship between the merging parties; (2) the relevant theory of harm or efficiency rationale; (3) the role of data, network effects, and ecosystem advantages; (4) the regulatory response and remedies adopted; (5) the balance between competition risks and potential efficiencies; and (6) lessons for Thailand’s digital merger-control framework. The purpose of these cases is not to classify all digital acquisitions as harmful, but to demonstrate how regulatory authorities can distinguish problematic consolidation from legitimate technology-driven integration.

A key illustration is the Grab–Uber merger in Southeast Asia, which demonstrates how platform consolidation can significantly alter market structure in multi-sided markets. Following the merger, Grab achieved dominant market positions in ride-hailing services across several ASEAN jurisdictions, raising concerns regarding network effects, price coordination, and entry barriers. The case also highlights heterogeneous regulatory responses across Singapore, Indonesia, and other ASEAN jurisdictions, reflecting differences in enforcement capacity and merger review frameworks.

Similarly, the Facebook–WhatsApp acquisition demonstrates how low-revenue targets can nevertheless hold significant strategic value due to user data and network effects. Although WhatsApp generated limited turnover at the time of acquisition, its large user base and data potential contributed to long-term competitive implications that were not fully captured through traditional turnover-based thresholds.

In addition, the Google–Fitbit merger illustrates the growing importance of data consolidation in digital markets. The transaction raised concerns about combining health data with existing advertising ecosystems, demonstrating that competitive harm may arise from data integration rather than price effects alone.

These cases collectively support the argument that merger control in digital markets must move beyond turnover-based and price-centric frameworks and incorporate forward-looking assessments of data control, innovation impact, and ecosystem effects.

## 8 Results/findings

The comparative analysis identifies five major findings regarding the regulation of digital-platform mergers and their implications for Thailand. Rather than evaluating jurisdictions according to a single regulatory model, the findings demonstrate that effective digital merger control depends on the interaction between notification design, substantive assessment tools, below-threshold review mechanisms, institutional capacity, and regulatory adaptability.

The first finding concerns the limitations of traditional notification systems based primarily on turnover thresholds. Turnover remains an important indicator of economic significance; however, it may not accurately reflect competitive importance in digital markets. Many digital start-ups generate limited revenue during early development stages while possessing significant assets in the form of user networks, data resources, algorithms, intellectual property, or innovation potential.

The comparative analysis shows different approaches to this challenge. The European Union continues to rely primarily on turnover-based jurisdictional thresholds but has developed sophisticated substantive assessment tools for evaluating digital competition concerns. China has retained turnover-based filing requirements while supplementing them with below-threshold review powers. Singapore’s voluntary notification system provides greater flexibility through case-by-case assessment, whereas Indonesia’s asset and turnover-based ex-post system faces greater difficulty in identifying potentially problematic acquisitions before market structures become established.

For Thailand, the key implication is that merger-control effectiveness depends not only on increasing or lowering thresholds but on designing notification mechanisms capable of identifying strategically significant digital acquisitions.

The second finding is that digital merger assessment requires broader evaluation criteria beyond traditional price-based competition analysis. Digital platforms frequently compete through service quality, innovation, privacy protection, user experience, data availability, and ecosystem integration rather than monetary price.

The EU and China demonstrate more developed approaches by incorporating factors such as data control, network effects, innovation incentives, and quality effects into merger assessment. Singapore similarly applies a flexible substantial-lessening-of-competition framework that allows consideration of non-price competitive effects.

The findings indicate that traditional tools such as SSNIP remain relevant but should not operate as the exclusive analytical framework in digital markets. Instead, merger review should incorporate complementary indicators, including SSNDQ analysis, data-access assessment, innovation competition, and ecosystem effects.

For Thailand, this suggests the need to strengthen substantive assessment capabilities rather than relying solely on existing market-share and turnover indicators.

The third finding relates to the difficulty of identifying acquisitions involving potential competitors. Digital firms may have limited current revenue but significant future competitive importance. This creates a regulatory gap where potentially important transactions may avoid review because they do not satisfy conventional notification requirements.

China provides an example of a more flexible approach through discretionary below-threshold review powers. Germany and other jurisdictions have introduced transaction-value mechanisms to capture acquisitions where the target’s strategic value exceeds its current financial performance. Conversely, the EU experience after the Illumina/GRAIL judgment demonstrates that even advanced systems face limitations when below-threshold review mechanisms are legally constrained.

For Thailand, the implication is that future reform should consider carefully designed supplementary mechanisms, such as transaction-value indicators or targeted call-in powers, while ensuring legal certainty and preventing unnecessary intervention in ordinary business transactions.

The fourth finding is that legal reform alone cannot ensure effective digital merger governance. Digital markets require competition authorities to possess expertise in economics, technology, data governance, and platform business models.

The comparative analysis demonstrates that regulatory effectiveness depends on institutional capacity to evaluate complex issues such as network effects, algorithmic advantages, ecosystem dependency, and innovation incentives. Advanced legal frameworks may still face limitations if authorities lack technical resources, while flexible systems may perform effectively when supported by strong institutional expertise.

Thailand’s challenge is therefore both legal and institutional. Strengthening merger review requires investment in specialist knowledge, analytical tools, cooperation with technology experts, and improved coordination with regional and international competition authorities.

The fifth finding is that Thailand should not directly copy foreign merger-control models but should adopt a context-specific hybrid approach. The EU provides lessons regarding substantive analysis of innovation and data-related effects. China demonstrates the value of flexible review mechanisms. Singapore highlights the importance of institutional adaptability. Indonesia and Malaysia illustrate challenges associated with limited merger-control flexibility.

The appropriate reform direction for Thailand is therefore a balanced framework combining improved notification mechanisms, digital-specific assessment criteria, and enhanced institutional capability. Such an approach would allow Thailand to address harmful digital concentration while maintaining incentives for investment, entrepreneurship, and technological development.

## 9 Discussion

The findings underscore a critical tension between traditional merger control frameworks and the structural realities of digital markets. Digital platforms compete not on price but on quality, innovation, data accumulation, and network effects, rendering conventional assessment tools insufficient. The SSNIP test, a cornerstone of traditional competition analysis, fails in zero-price markets because it cannot measure hypothetical price increases where no monetary price exists. In contrast, the SSNDQ test offers a more suitable metric by assessing quality reductions, a key vector of harm in digital ecosystems.

This interpretation is strongly supported by the industrial organization literature on digital markets, which emphasizes that competition in platform ecosystems is primarily driven by innovation, data accumulation, and network effects rather than static price competition (Cabral et al., 2021). Recent studies show that acquisitions in digital markets may reduce innovation incentives and eliminate potential future competitors even when immediate price effects are negligible. In particular, the literature on “killer acquisitions” highlights that incumbent platforms may acquire nascent competitors to prevent future competitive threats rather than to achieve short-term efficiency gains. Furthermore, theoretical work demonstrates that merger control policies may influence firms’ innovation incentives and acquisition strategies, suggesting that regulatory design must account for dynamic efficiency effects rather than static welfare measures alone.

The divergence in institutional sophistication between advanced and emerging jurisdictions is pronounced. The EU’s evolving practice, including its increased use of Article 22 referrals and attention to data-driven entry barriers, reflects an advanced and adaptive regulatory environment. China’s rapid institutional development, marked by sector-specific guidelines and consideration of data as a competitive asset, demonstrates significant progress in regulating digital mergers.

In contrast, many emerging economies remain constrained by limited resources, rigid legal frameworks, or insufficient analytical capacity. The absence of merger control in Malaysia, the high thresholds in Indonesia, and the traditional price-centric framework in Thailand illustrate systemic vulnerabilities. These weaknesses create regulatory blind spots that large digital incumbents may exploit to consolidate market power through acquisitions of nascent rivals.

The global trend toward transaction-value thresholds, market-value criteria, and ex-post review mechanisms indicates a shift away from static, turnover-based triggers toward dynamic, innovation-sensitive regulatory models. International institutions such as the OECD and UNCTAD consistently emphasize that modern merger control must account for potential competition, future innovation trajectories, and the strategic significance of user data.

Overall, the discussion highlights the urgent need for emerging economies to recalibrate their merger control systems to reflect the competitive dynamics of the digital age. Without such reforms, these jurisdictions risk systemic under-enforcement and long-term market distortions.

## 10 Conclusion

The comparative assessment in this paper highlights that digital markets demand merger control frameworks capable of addressing data-driven competition, multi-sided platforms, and zero-price models, as traditional tools like turnover thresholds and the SSNIP test are increasingly inadequate. Experiences from China and the EU show that effective regulation requires non-price competitive factors, data-access considerations, innovation-focused indicators, and the ability to review below-threshold transactions. Emerging economies, including Thailand, face challenges due to limited institutional capacity, analytical tools, and legal flexibility. ASEAN jurisdictions exhibit varying readiness: Singapore employs flexible substantive tests, Indonesia applies ex-post notifications, and Malaysia is still developing general provisions. OECD and UNCTAD guidelines emphasize transaction-value thresholds, ex-post review, and cross-border cooperation. For Thailand specifically, the findings indicate that reform should be gradual and institutionally realistic. Rather than adopting foreign models directly, Thailand should develop a hybrid framework combining improved notification mechanisms, digital-specific assessment criteria, and enhanced enforcement capacity. The objective should be to create a merger-control system capable of identifying harmful digital consolidation while preserving innovation incentives and supporting legitimate technology-driven growth. To safeguard competitiveness and innovation, reforms in emerging economies should integrate data-centric tools, alternative thresholds, below-threshold review mechanisms, non-price effects, and stronger institutional cooperation aligned with global best practices.

## Data Availability

The original contributions presented in the study are included in the article/supplementary material, further inquiries can be directed to the corresponding author/s.

## References

[ref1] Albshaier L. Almarri S. Hafizur Rahman M. M. (2024). A review of blockchain’s role in E-commerce transactions: open challenges, and future research directions. Computers 13:27. doi: 10.3390/computers13010027

[ref2] Benkert B. J. M. Letina I. Liu S. (2025). *Acquihire: what Economics ℡ls Regulators*. Antitrust Chronicle. Available online at: https://www.igorletina.com/files/2025_Benkert_Letina_Liu_AntitrustChronicle.pdf.

[ref3] Bourreau M. De Streel A. (2020). *Big Tech Acquisitions*. Centre on Regulation in Europe Available online at: https://cerre.eu/wp-content/uploads/2020/03/cerre_big_tech_acquisitions_2020.pdf.

[ref4] Bryan K. A. Hovenkamp E. (2020). Startup acquisitions, error costs, and antitrust policy. U. Chi. L. Rev. 87, 331–356. Available online at: https://chicagounbound.uchicago.edu/uclrev/vol87/iss2/3

[ref5] Cabral L. M. B. Haucap J. Parker G. Petropoulos G. Valletti T. M. Van Alstyne M. W. (2021). *The EU Digital Markets Act: A Report from a Panel of Economic Experts*. SSRN Scholarly Paper No. 3783436. Social Science Research Network Available online at: https://papers.ssrn.com/abstract=3783436.

[ref6] Choe C. Shekhar C. (2010). Compulsory or voluntary pre-merger notification? Theory and some evidence. Int. J. Ind. Organ. 28, 10–20. doi: 10.1016/j.ijindorg.2009.05.001

[ref7] Eduardo H. Anzani E. Natalia T. S. (2025). A comparative analysis of KPPU sanctions on tender collusion in public procurement in Indonesia. Just. Sains 10, 1–17. doi: 10.24967/jcs.v10i1.3572

[ref8] Fast V. Schnurr D. Wohlfarth M. (2023). Regulation of data-driven market power in the digital economy: business value creation and competitive advantages from big data. J. Inf. Technol. 38, 202–229. doi: 10.1177/02683962221114394

[ref9] Gürkaynak G. Dalkır S. Durlu D. (2014). Emerging markets and US horizontal merger guidelines: a Turkish competition law perspective. J. Compet. Law Econ. 10, 445–474. doi: 10.1093/joclec/nht037

[ref10] Hemphill C. S. Wu T. (2020). Nascent competitors. Univ. Pa. Law Rev. 1, 1879–1910. doi: 10.2139/ssrn.3624058

[ref11] Hofman B. Petry J. (2025). *Internationalization of the RMB: Status, Options and Risks*. China Kennisnetwerk. CKN Report Internationalization of the RMB.

[ref12] Illumina Inc. and Grail Inc. European Commission, ECLI:EU:C:2024:677 ___ (Court of Justice 2024). (2024). Available online at: https://www.wsgr.com/en/insights/illuminagrail-eu-court-overturns-below-threshold-merger-review-policy.html.

[ref13] Iwuji A. E. R. (2025). *The Main Purposes and Principles of the eu Merger Control System*. Vilniaus Universitetas.

[ref14] Jensen M. C. (2010). Value maximization, stakeholder theory, and the corporate objective function. J. Appl. Corp. Financ. 22, 32–42. doi: 10.1111/j.1745-6622.2010.00259.x

[ref15] Kaplow L. (2021). Horizontal merger analysis. Int. J. Ind. Organ. 79:102714. doi: 10.1016/j.ijindorg.2021.102714

[ref16] Katagar S. S. (2024). Digital merger and its challenges to the competition law: Indian laws. LawFoyer Int J. Doctrinal Legal Rsch. 2:43. Available online at: https://lijdlr.com/2024/06/06/digital-merger-and-its-challenges-to-the-competition-law-indian-laws/

[ref17] Koirala S. Rao S. Farag H. Marshall A. (2023). The market for corporate control and risk-taking: evidence from global merger and acquisition laws. Br. J. Manag. 34, 997–1022. doi: 10.1111/1467-8551.12625

[ref18] Lee C. (2014). Competition law enforcement in Malaysia: some recent developments. Malays. J. Econ. Stud. 51, 77–88. Available online at: https://mjes.um.edu.my/index.php/MJES/article/view/2880

[ref19] Lee J. W. (2023). Data-driven mergers: is it time to reform EU merger control? De Lege Ferenda 6:103. Available online at: https://www.cambridgelawreview.org/issues-dlf-volume-vi

[ref20] Lee Y. Geum Y. (2023). Identifying patterns of mergers and acquisitions in startup: an empirical analysis using crunchbase data. IEEe Access 11, 42463–42472. doi: 10.1109/access.2023.3270623

[ref21] Lindeboom J. (2025). Illumina/Grail: flawed originalism and the judicial hunch. J. Antitrust Enforc. 13, 223–232. doi: 10.1093/jaenfo/jnaf007

[ref23] Mandrescu D. (2018). The SSNIP test and zero-pricing strategies: considerations for online platforms. Eur. Competition & Reg. L. Rev. 2:244. doi: 10.21552/core/2018/4/4

[ref24] Mommers M. Landry A. (2024). The FTC & DOJ’S new merger guidelines: a new path or more of the same? Emory Corp. Gov. Account. Rev. 11:205. Available online at: https://scholarlycommons.law.emory.edu/ecgar/vol11/iss2/7/

[ref25] Nademi N. (2025). *The Hypothetical Monopolist Test Expanded-Quality Considerations through SSNDQ and SSNIC*.

[ref26] Ong B. Toh D. J. (2023). Digital dominance and social media platforms: are competition authorities up to the task? IIC Int. Rev. Intellect. Property Competition Law 54, 527–572. doi: 10.1007/s40319-023-01302-1, 37073397 PMC10031722

[ref27] Pantazatou K. Kafteranis D. Finelli F. (2025). Participatory enforcement in combating economic crimes: insights from tax offences, money laundering and sanctions evasion. Eur. Law J. 31, 153–176. doi: 10.1111/eulj.70011

[ref28] Parker G. Petropoulos G. Van Alstyne M. W. (2020). “Digital Platforms and Antitrust,” in The Oxford Handbook of Institutions of International Economic Governance and Market Regulation, eds. Brousseau E. Glachant J. M. Sgard J. (Oxford: Oxford University Press).

[ref29] Pehlivanli A. S. (2025). From Consumer Welfare to Fairness: A Functional Framework for Controlling Digital Markets and beyond under EU Competition Law. Durham: Durham University.

[ref30] Qihoo 360 and Tencent. (2013). *Min san Zhong Zi no. 4 (supreme people’s court October 16, 2014)*. Available online at: https://www.wipo.int/wipolex/en/text/578092.

[ref31] Qin V. Pauwels K. Zhou B. (2024). Data-driven budget allocation of retail media by ad product, funnel metric, and brand size. J. Mark. Anal. 12, 235–249. doi: 10.1057/s41270-024-00294-2

[ref32] Rahman N. A. Tupari M. A. Ahamat H. (2024). Merger control regime in Malaysia: past, present and way forward. IIUMLJ 32:118. doi: 10.31436/iiumlj.v32i2.998

[ref33] Sidak J. G. Teece D. J. (2009). Dynamic competition in antitrust law. J. Competition Law Econ. 5, 581–631. doi: 10.1093/joclec/nhp024

[ref300] State Council of the People’s Republic of China. (2018). Provisions of the state council on the notification threshold for concentrations of undertakings [国务院关于经营者集中申报标准的规定] (promulgated 3 August 2008, amended 18 September 2018).

[ref34] Sukarmi S. Tejomurti K. Silalahi U. (2024). Digital market and its adequacy of merger assessment in Indonesian business competition law. Int. J. Law Manag. 66, 694–719. doi: 10.1108/IJLMA-08-2023-0185

[ref35] Tian L. Wang D. (2026). More transparency, more compliance? Digital government services and corporate tax avoidance. Econ. Gov. 27:18. doi: 10.1007/s10101-026-00360-8

[ref36] Trade Competition Act B.E. 2560 (2017), §§ 51–52. (2017). *OTCC official English PDF*. Available online at: https://www.asean-competition.org/file/pdf_file/Thailand%20Trade%20Competition%20Act%202017.pdf.

[ref37] Tzanaki A. (2024). *Illumina’s Light on Article 22 EUMR: the Suspended Step and Uncertain Future of EU Merger Control Over Below-Threshold “Killer” Mergers*. Competition Policy International-CPI Antitrust Chronicle on" Illumina/GRAIL.

[ref38] U.S. Department of Justice Antitrust Division and Federal Trade Commission. (2023). *Merger Guidelines (Section 2.9)*. Available online at: https://www.justice.gov/atr/merger-guidelines.

[ref39] Valiyeva A. (2024). *Big Data Mergers in the European Union: Regulatory Responses to Data-Related Concerns*.

[ref40] Völcker S. B. (2024). Back to the future: merger control outside the merger regulation. Common Market Law Rev. 61, 1223–1254. doi: 10.54648/cola2024080

[ref41] Wang D. Hu J. Ma S. (2026). Data-driven empowerment: how government data governance facilitates firm upgrading. Eng. Econ. 37, 106–122. doi: 10.5755/j01.ee.37.1.41789

[ref42] Xiong Q. Deng F. Li H. Xie D. (2025). Optimizing the pre-merger notification rule with regulatory data: empirical analysis from China. J. Asian Econ. 101:102057. doi: 10.1016/j.asieco.2025.102057, 38826717

[ref43] Zakaria M. R. A. Khan M. B. A. Rahman N. A. Yusof A. M. (2025). Jurisdictional overlap between the Malaysian competition commission and the securities commission in merger review. IIUMLJ 33:1. doi: 10.31436/iiumlj.v33i1.1054

[ref44] Zou X. Wang Z. Zhao J. (2025). The impact of ESG performance of acquirer on the long-term performance of cross-border mergers and acquisitions of China A-share listed companies: an analysis based on two-way fixed effect and threshold effect. Sustainability 17:6566. doi: 10.3390/su17146566

